# Advances in large DNA fragment assembly for microbial cell factory engineering

**DOI:** 10.1002/qub2.70039

**Published:** 2026-05-06

**Authors:** Yu Zhang, Wenqian Liu, Dongyuan Cheng, Jiazhang Lian, Xian Fu, Yue Shen

**Affiliations:** ^1^ Key Laboratory of Biomass Chemical Engineering of Ministry of Education & State Key Laboratory of Biobased Transportation Fuel Technology College of Chemical and Biological Engineering Zhejiang University Hangzhou China; ^2^ State Key Laboratory of Genome and Multi‐omics Technologies BGI Research Hangzhou China; ^3^ BGI Research Changzhou China; ^4^ Guangdong Provincial Key Laboratory of Genome Read and Write BGI Research Shenzhen China

**Keywords:** in vitro and in vitro assembly, large DNA fragment assembly, microbial cell factories, neochromosome, synthetic genome

## Abstract

The efficient, rapid, and reliable assembly of DNA fragments is essential for advancing metabolic engineering and synthetic biology. With the rapid advancement of DNA synthesis and assembly technologies, the scale of DNA assembly has expanded from single genes to metabolic pathways and even genomes. Large DNA fragment assembly, in particular, has developed into a pivotal tool for microbial cell factory engineering, significantly contributing to the understanding and construction of biological systems. This review summarizes recent advancements in large DNA fragment assembly methods, encompassing in vitro and in vitro methods for multiple‐gene assemblies, as well as genome‐scale technologies, including synthetic genome and neochromosome construction. We systematically compare their key features in terms of assembly principle, capacity, and efficiency. Especially, we highlight their applications in microbial cell factory engineering, including heterologous pathway construction, reprogramming host metabolism, and expanding complex biosynthetic networks. Finally, we discuss the challenges and prospects of applying large DNA fragment assembly to advance cell factories. In summary, this review provides a theoretical and technical framework for engineering high‐performance microbial cell factories, contributing to the advancement of industrial biomanufacturing.

## 1 INTRODUCTION

The construction of microbial cell factories has revolutionized industrial biomanufacturing, enabling sustainable production of high‐value compounds, including pharmaceuticals, biofuels, and fine chemicals [1]. Building efficient microbial cell factories typically involves: (1) introducing heterologous metabolic pathways larger than 10 kilobases (kb) (e.g., incorporating dozens of plant‐, bacterial‐, and fungal‐derived genes to produce alkaloids, taxol, and other natural products [2, 3, 4]); (2) reprogramming host metabolism at the genome scale (megabase [Mb] level); and (3) expanding complex biosynthetic networks (often requiring DNA constructs spanning hundreds of kb to Mb [5, 6, 7]). These requirements highlight the urgent need for advanced DNA assembly technologies, especially large DNA fragment (ranging in length from tens of kb to Mb) assembly. This technological capability serves as a foundation for engineering microbial cell factories in the aforementioned aspects.

The large DNA fragment is assembled through a hierarchical splicing strategy using synthetic short oligonucleotides (oligos), which relies heavily on low‐cost and large‐scale oligo synthesis [8]. Recent advances in DNA synthesis technologies have significantly promoted DNA synthesis quality, throughput, efficiency, and cost reduction. For example, high‐density, integrated chip‐based DNA synthesis technologies have cut costs and increased throughput exponentially [8, 9, 10]. Oligo assembly technologies enable the assembly of short oligos into gene‐length double‐stranded DNA (dsDNA) (kb level) [11]. Engineered terminal deoxynucleotidyl transferases further enable longer, high‐fidelity single‐stranded DNA (ssDNA) synthesis [12, 13]. These improvements provide higher‐quality and more cost‐effective materials for large DNA fragment assembly, thereby substantially advancing the development of DNA assembly methodologies.

Over the past two decades, large DNA fragment assembly has improved in length, accuracy, speed, etc., and a proliferation of novel methods has emerged—spanning in vitro and in vitro assembly, synthetic genomes, and neochromosome (NeoChr) assembly—providing solid technical support for the engineering of microbial cell factories (Figure 1A,B). As summarized in Table 1, traditional and emerging in vitro methods are increasingly excelling in combining standardized and modularized biological parts, transcription units (TU), and multigene pathways [67]. However, due to the instability of in vitro manipulation for large DNA fragments, stable assembly of longer sequences—particularly natural product biosynthetic pathways or biosynthetic gene clusters (BGCs) ranging from 10 kb to Mb—still relies heavily on in vitro assembly approaches [68, 69]. Recent advancements, such as *Escherichia coli* (*E*. *coli*) genome recoding and the Synthetic Yeast Genome Project (Sc2.0) of *Saccharomyces cerevisiae* (*S*. *cerevisiae*), have expanded the assembly capability to the genome scale [46, 48], facilitating genome‐wide strain engineering. Further innovations, such as the de novo synthesis of supernumerary, orthogonal NeoChrs, push assembly boundaries even further and enable the expansion of metabolic networks [6]. Despite these breakthroughs in large DNA fragment assembly technology, comprehensive reviews on their applications in microbial cell factory development remain limited.

**FIGURE 1:**
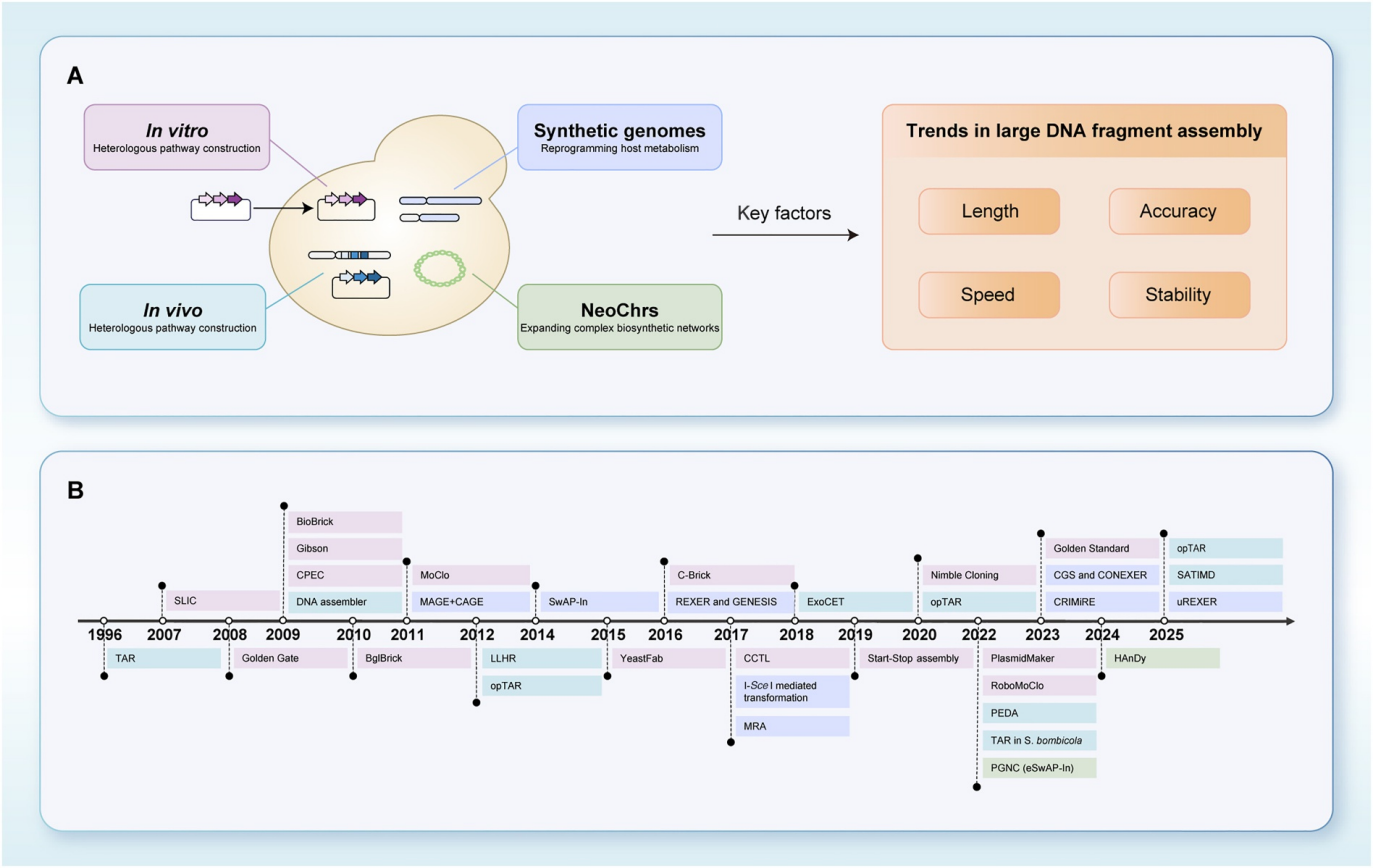
Overview of large DNA fragment assembly technologies in microbial cell factories. (A) Large DNA fragment assembly facilitates microbial host engineering in pathway construction, metabolism reprogramming, and network expansion, with current development trends focusing on some key factors. (B) Technological timeline highlights key milestones in large DNA fragment assembly.

**TABLE 1:** DNA assembly methods, with a focus on methods with applications in biosynthesis.

Category	Host	Assembly method^a^	Size/number^b^	Key features	Applications of microbial cell factory engineering
In vitro DNA assembly	No limitation	BioBrick [14, 15]	20 kb/2 fragments	Modular assembly with Type IIP restriction enzymes	Assembly of biological parts, TUs, and multigene pathways
		BglBrick [16]	2 fragments	Reliable: *Bgl* II/*BamH* I cutters with high efficiency.	Enables the construction of gene expression devices, chimeric protein fusions, and targeted genomic integrations in *E. coli*
				Methylation‐insensitive: Works despite dam/dcm methylation.	
				Benign scar: 6 bp glycine–serine linker.	
		Golden Gate [17]	30 kb	Modular assembly with Type IIS restriction enzymes	Assembly of biological parts, TUs, and multigene pathways
		Start‐Stop assembly [18]	15 fragments	Scarless translation junctions; allowing assembly of up to 15 genes from up to 60 parts	Applied for constructing combinatorial carotenoid biosynthetic libraries in *E*. *coli*
		MoClo [19, 20]	33 kb/11 TUs	Stepwise, modular construction of multigene pathways; allowing the construction of 11 transcriptional units from 44 individual modules in just three cloning steps	Assembly of biological parts, TUs, and multigene pathways
		YeastFab [21, 22]	6 fragments	Modular DNA assembly framework for constructing multigene pathways in yeast; 11 POT modules enable assembly of a 6‐gene pathway; a 151‐promoter library enables 3‐gene expression optimization	Combinatorial assembly for optimizing β‐carotene production
		Golden Standard [23]	20 TUs	6 constitutive + 4 inducible promoters; 4 mono + 2 bicistronic RBSs; 7 N‐terminal + 5 C‐terminal tags; 2 CDS types; 6 terminators; 15 linkers; 3 transcription factors; 41 host vectors.	Optimizing the biosynthetic pathway for β‐carotene production and the expression of recombinant proteins
		RoboMoClo [24]	8 TUs	Robotics‐assisted modular cloning	Lycopene biosynthesis in *Corynebacterium glutamicum*
		Gibson [25]	900 kb	A one‐pot, isothermal approach for joining multiple overlapping DNA fragments	Seamlessly constructing synthetic and natural genes, genetic pathways, and entire genomes
		Nimble Cloning [26]	3 fragments	*Sfi* I/T5 for linearization and overhangs; standardized adapters for primers; more flexible and higher cloning efficiency than Gibson	Supports both PCR products and entry clones
		SLIC [27]	10 fragments	Uses 3'→5' exonuclease to create single‐stranded overhangs, enabling annealing of complementary DNA fragments	Multifragment assembly
		CPEC [28]	8.4 kb	Employs a polymerase‐mediated extension of overlapping DNA fragments to generate circular, seamless constructs in a single reaction	Assembly and cloning of complex combinatorial libraries and multipart pathways
		C‐Brick [29]	2 fragments	Uses Cas12a (Cpf1)‐generated 4–5 bp 5′ overhangs for directional, modular assembly of multiple DNA fragments; assembly efficiency up to 29.4%, accuracy up to 93.6%	Assembled chromoprotein cassettes for pigment expression in *E*. *coli*
		CCTL [30]	‐	Combines Cas12a (Cpf1)‐assisted cleavage with Taq DNA ligase for high‐precision ligation of large DNA fragments; assembly efficiency up to 78% and accuracy 100% in high‐GC regions	Actinorhodin production in *Streptomyces*
		PlasmidMaker [31]	27 kb/11 fragments	101 plasmids (5–27 kb) from up to 11 fragments were assembled across six species with minimal human intervention, handling GC contents up to 77%; accuracy ranging from 18% to 90%	High‐throughput assembly of large plasmids with multiple fragments and high GC content
In vivo DNA assembly	*E. coli*	LLHR [32, 33]	52 kb	The full‐length RecET system (LLHR) exhibits 20‐ and 40‐fold higher efficiency than its truncated counterpart and the red system, respectively	Direct cloning of 10 megasynthetase gene clusters from *P*. *luminescens*; identification of a putative colletochlorin BGC in *Colletotrichum higginsianum*
		ExoCET [34]	106 kb	Direct cloning efficiency has been increased by up to 32‐fold, enabling one‐step cloning of BGCs larger than 100 kb	Direct cloning of the 106‐kb salinomycin BGC from *S*. *albus*
	*S. cerevisiae*	TAR [35]	600 kb	TAR avoids the limitations of in vitro cloning methods in terms of length and cloning BGCs from complex backgrounds	Direct cloning of large human DNA
		opTAR [36]	454 kb	Enhanced stability of assemblies via the addition of yeast origins of replication (the maximum size improved from 142 to 454 kb)	Assembly and stable maintenance of the 454‐kb genomic DNA from *Synechococcus elongatus* PCC 7942
		opTAR [37]	67 kb	Enhanced efficiency of BGC cloning via K1α selection (from 0.26% to 13%)	Cloning of the 35‐kb chelocardin and 67‐kb daptomycin biosynthetic gene clusters from *Amycolatopsis sulphurea* and *Streptomyces filamentosus*
		opTAR [38]	76 kb	Enhanced efficiency of BGC cloning via hook optimization (a 70% capture rate for corbomycin BGC)	Cloning of the 76‐kb corbomycin BGC from *Streptomyces* sp. WAC01529
		DNA assembler [39, 40, 41]	45 kb	Rapid and highly efficient (70%–100%) assembly of multigene pathways	Assembly of three distinct pathways: a 9‐kb (3‐gene) D‐xylose utilization pathway, an 11‐kb (5‐gene) zeaxanthin biosynthetic pathway, and a combined 19‐kb (8‐gene) pathway for both functions
	*Aspergillus nidulans*	TAR [42]	‐	Enhanced efficiency (about 70% fusion rate with 25 bp overlaps) and capacity (one‐step assembly of 6 fragments) via NHEJ deficiency	Streamlined construction of self‐replicating plasmids lacking bacterial sequences
	*Starmerella bombicola*	TAR [43]	‐	CRISPR/Cas9‐mediated one‐step in vivo assembly	Assembly of multiple DNA fragments (10 kb) for acid‐type sophorolipid production
	*Trichoderma reesei*	SATIMD [44]	32.7 kb	Targeted integration and stitching efficiencies are close to 100% and over 80%, respectively	Assembly of a 32.7‐kb sorbicillinoid BGC (up to 10 different DNA fragments)
	*E*. *coli*, *Ralstonia eutropha*, *Pseudomonas putida*, *Lactobacillus plantarum*, and *Yarrowia lipolytica*	PEDA	6 kb	Assembly efficiency is more than double that of wild‐type *E*. *coli* lacking T4 ligase and T5 exonuclease	RBS library construction in *P*. *putida* and direct pathway assembly into the production strain
Synthetic genome assembly	*E*. *coli*	MAGE and CAGE [7]	2.3 Mb	MAGE enables scattered, genome‐scale editing but introduces a high frequency of off‐target mutations; CAGE’s reliance on iterative conjugation and selection makes large‐fragment assembly a time‐consuming process (weeks to months)	Assembly of a UAG stop codon‐free *E*. *coli* genome for ncAA incorporation and antiviral properties
		REXER and GENESIS [45]	0.5 Mb	Long synthetic DNA is capable of replacing the genome stepwise; the iterative marker‐swapping process, utilizing CRISPR/Cas9 and λ‐Red recombination, enables precise and efficient assembly; dependence on electroporation to introduce fragments yields low efficiency	Assembly of the recoded genome strain Syn61 for the production of noncanonical heteropolymers and macrocycles and for broad‐spectrum phage resistance
		uREXER [46]	0.1 Mb	The streamlined REXER variant uses universal CRISPR/Cas9 spacers to enable more efficient 100 kb fragment replacement	Assembly of the recoded genome strain Syn57
		CGS and CONEXER [47]	0.5–1.1 Mb	Coupling REXER with bacterial conjugation decreases the time for a single integration from 4 days to 1 day; RecA knockout minimizes crossovers between the synthetic DNA and the genome, without the need for sequencing	‐
	*S*. *cerevisiae*	SwAP‐In [48, 49, 50, 51, 52, 53, 54]	0.18–0.75 Mb	Coupling yeast HR with dual auxotrophic selection ensures high‐fidelity assembly; this stepwise megachunk integration is time‐consuming (5 days for single integration); the assembly efficiency decreases as the number of rounds extends	Assembly of synthetic yeast chromosomes synI, synIII, synV, synVI, synIX, synX, and synXIV for conditional tolerance and production improvement
		I‐*Sce* I‐mediated transformation [55, 56, 57]	0.77–1.03 Mb	By performing two halves of the assembly in parallel, it is about 50% faster than SwAP‐In	Assembly of synthetic yeast chromosomes synII, synVII, and synXIII for production improvement
		MRA [58, 59, 60, 61]	0.51–1.45 Mb	Parallel assembly of multiple segments across several haploid strains dramatically reduces the timeline compared to sequential methods; meiotic recombination carries a high risk of crossover with wild‐type sequences	Assembly of synthetic yeast chromosomes synIV, synVIII, synXII, and synXVI for conditional tolerance
		CRIMiRE [62, 63]	0.66–1.05 Mb	Using targeted CRISPR/Cas9 cleavage to avoid the random cross‐mixing of MRA improves assembly fidelity	Assembly of synthetic yeast chromosomes synXI and synXV
NeoChr assembly	*S*. *cerevisiae*	TAR [64]	100 kb	Successful large‐scale assembly (44 fragments, 100‐kb NeoChr) with high fidelity (36%)	NeoChrs as an exclusive expression platform for the swapping of the glycolytic pathway
		TAR [5, 65]	100 kb	Higher assembly efficiency of circular chromosomes compared to linear chromosomes (36% vs. 0% faithful to the original design)	The integration of the glycolysis, pentose phosphate, bacterial vanillic acid, and plant anthocyanin synthesis pathways onto NeoChrs enabled the de novo biosynthesis of anthocyanin
		PGNC/eSwAP‐In [66]	211 kb	Modular assembly (21 chunks); superior growth rate and stability of circular versus linear chromosomes	PGNC enabled carbon source expansion and enhanced phenotypic diversity via the SCRaMbLE system
		HAnDy [6]	1.024 Mb	High efficiency (about 60%) and superior genetic stability	NeoChrs enhance host metabolism (9 new compounds) and environmental adaptability

^a^ Representative methods and new methods developed in the last 5 years.

^b^ “‐” indicates “data not shown.”

In this review, we systematically summarize recent advances in large DNA fragment assembly technologies, focusing on their applications in microbial cell factory engineering, with particular emphasis on industrial workhorses (e.g., *E*. *coli* and *S*. *cerevisiae*). From in vitro assembly to in vitro assembly and further to synthetic genome assembly and supernumerary designer NeoChr construction, we introduce their key features in terms of assembly principle, capacity, and efficiency and highlight their respective strengths in microbial cell factory engineering. Finally, we discuss the current limitations and future directions in the application of large DNA fragment assembly technology.

## 2 IN VITRO DNA ASSEMBLY METHODS FOR HETEROLOGOUS PATHWAY CONSTRUCTION

In vitro DNA assembly has become a central tool for reconstructing heterologous metabolic pathways in microbial systems (Figure 2A). The complexity of large DNA constructs necessitates efficient assembly to maintain stability and improve construction throughput [70]. Restriction digestion‐based cloning established standardized frameworks for modular pathway construction, yet its reliance on specific recognition sites limited sequence flexibility. Homology‐directed assembly enabled seamless and flexible fragment joining, overcoming sequence limitations. The emergence of programmable nuclease‐guided systems and automated platforms has further enhanced precision and scalability, advancing the construction of complex biosynthetic pathways.

**FIGURE 2:**
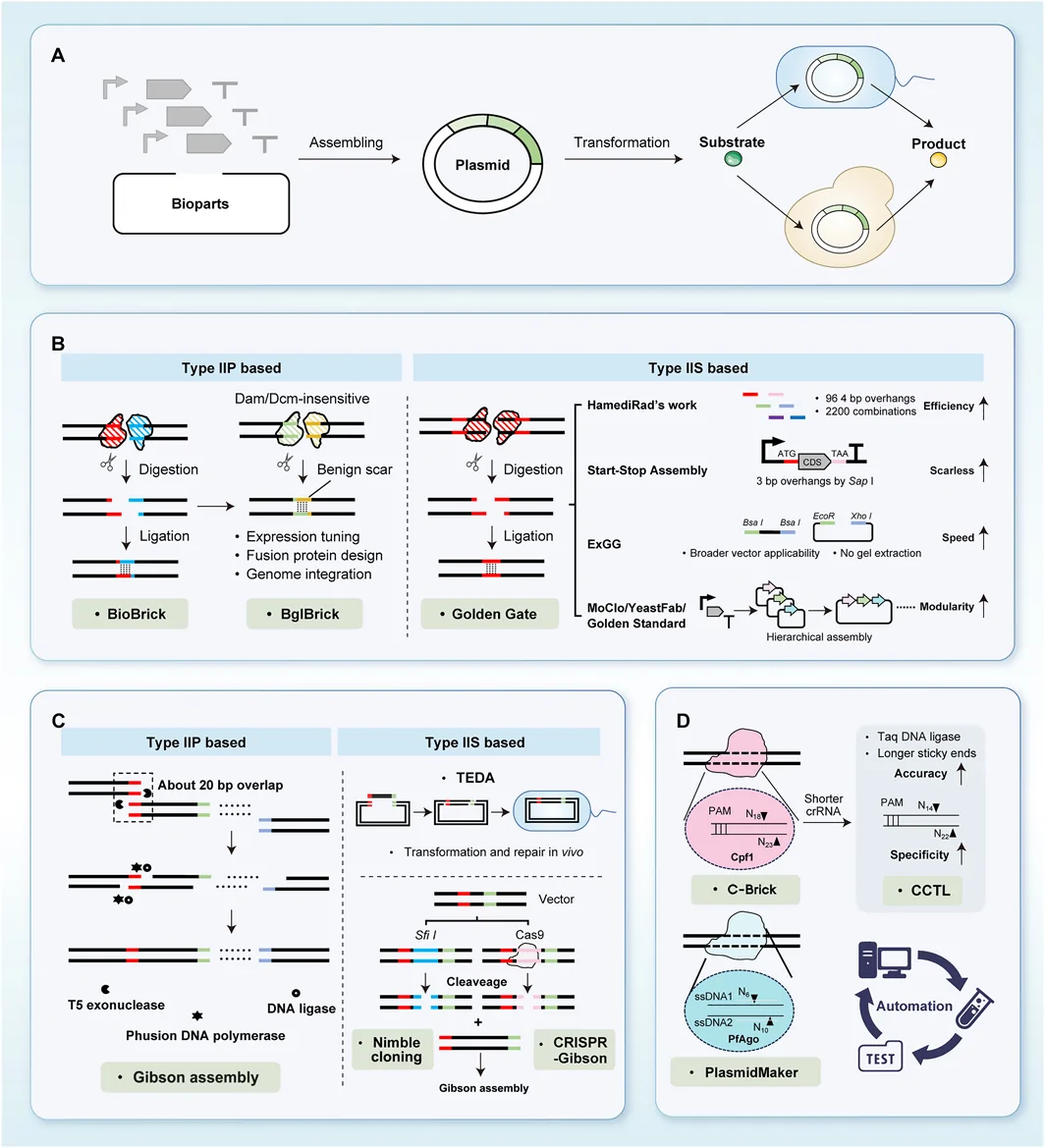
Development and applications of in vitro large DNA fragment assembly. (A) In vitro assembly of large DNA fragments enabling heterologous pathway construction in microbial cell factories. Principles and development trends of (B) restriction digestion‐based, (C) homologous recombination‐based, and (D) programmable nuclease‐guided assembly methods.

### 2.1 Restriction digestion‐based methods for standardized and modular pathway construction

Restriction digestion‐based DNA assembly remains a foundational approach for constructing modular genetic circuits and metabolic pathways. By exploiting the sequence‐specific cleavage properties of Type II restriction endonucleases, researchers can systematically join genetic elements in a predictable and reusable manner, enabling standardized design of multigene constructs for microbial cell factories [71]. Early systems, such as the BioBrick assembly (Figure 2B), established a framework for modular cloning (MoClo), defining fixed junction sequences between DNA parts to ensure interoperability and reusability [14]. To overcome limitations associated with the scar sequences and directional constraints of the original standard, BglBrick was developed (Figure 2B). This system employs *Bgl* II and *BamH* I restriction enzymes, which generate a 6‐nucleotide scar encoding a glycine‐serine linker, a benign peptide that does not interfere with most protein fusions [16]. This design ensures directional assembly, compatibility with existing BioBrick parts, and stepwise ligation of multiple fragments without introducing unwanted sequences. Through standardized MoClo, BioBrick‐based methods have been applied to the assembly of polyketide synthase (PKS) pathways [15] and the biosynthesis of amino acids and cofactors [72, 73], facilitating the modular engineering of microbial metabolic pathways. However, the reliance on fixed restriction sites limits multigene assembly, as only stepwise two‐ or three‐fragment ligations are possible [16, 67], making BioBrick‐based construction time‐consuming and less efficient for large or complex pathways.

By contrast, Type IIS enzymes such as *Bsa* I and *BsmB* I cleave DNA outside of their recognition sites to generate user‐defined overhang pairs, enabling single‐enzyme, directional, and multifragment assembly. It overcomes the fixed‐site limitations of Type IIP enzymes, allowing efficient multigene pathway construction in microbial cell factories. As shown in Figure 2B, Golden Gate assembly (GGA) enables one‐pot construction of multiple DNA fragments, with successful assembly of up to 11 fragments reported [17, 74]. In practical applications, GGA has enabled the synthesis of complex metabolites in yeast, such as archeal‐eukaryotic hybrid lipids [75] and more than 200 diterpenes [76], showcasing its broad utility and potential in metabolic engineering. Recent advances have optimized overhang sequences to maximize efficiency. HamediRad et al. have screened over 200 overhang combinations and demonstrated near 100% assembly efficiency for 3–5 fragment constructs using iBioCAD, underscoring the enhanced accuracy and reliability enabled by overhang optimization [77]. Additionally, Start‐Stop assembly relies on *Sap* I to generate 3 bp overhangs encoding start and stop codons, enabling seamless assembly of coding sequences while preserving mRNA secondary structure and ribosome binding site (RBS) activity, supporting high‐efficiency multipart assembly and modularity [18]. Complementing these strategies, Expanded Golden Gate takes advantage of *Bsa* I‐generated overhangs that are compatible with the multiple cloning site sequences of standard vectors, allowing PCR products to be ligated in a defined orientation and simplifying cloning design [78]. Collectively, these innovations reduce assembly errors, maintain functional integrity of pathway genes, and streamline the construction of heterologous metabolic modules, directly supporting efficient design and testing in microbial cell factories (Figure 2B).

Beyond junction‐level optimizations, hierarchical assembly strategies have enabled the construction of increasingly large and complex heterologous pathways. MoClo exemplifies this approach, employing *Bsa* I and *Bpi* I to generate customizable overhangs that allow precise, directional assembly of up to eight DNA modules simultaneously [19]. Its hierarchical design allows stepwise construction—from basic bioparts to TUs and further to multigene pathways—efficiently building constructs up to 33 kb in just three steps [20]. Similar hierarchical strategies have been applied in GoldenBraid [79] for plants and YeastFab [21, 22] for yeast, facilitating modular pathway construction in more hosts. In addition, the Golden Standard adopts the Standard European Vector Architecture and provides efficient, standardized genetic tools for α‐, β‐, γ‐, and δ‐proteobacteria [23]. These platforms allow systematic assembly and combinatorial testing of metabolic modules, supporting rapid exploration of pathway designs and optimization of enzyme expression in different microbial backgrounds.

### 2.2 Homologous recombination‐based methods for seamless and flexible pathway assembly

Unlike restriction enzyme‐based approaches, homologous recombination‐based (HR‐based) approaches do not rely on predefined recognition sites, allowing precise and seamless joining of multiple fragments regardless of their internal sequences (Figure 2C). Gibson assembly exemplifies this capability, employing T5 exonuclease, DNA polymerase, and DNA ligase in a single isothermal reaction, which enables assembly of multiple fragments, including constructs exceeding 100 kb, in a single step [25, 80]. The seamless nature preserves coding sequences and regulatory elements, maintaining the functional integrity of open reading frames, which is an essential feature for constructing heterologous metabolic pathways. Gibson assembly has been successfully applied to the assembly of large human *IGHV* gene fragments [81] and modular genetic circuit construction [82]. T5 exonuclease‐dependent assembly only employs T5 exonuclease to generate short single‐stranded overhangs that allow homologous DNA fragments to anneal seamlessly. The gaps are subsequently repaired in vivo, enabling simple and efficient multifragment assembly without complex enzyme mixtures [83]. Other HR‐based strategies, such as sequence and ligation‐independent cloning (SLIC) [27] and circular polymerase extension cloning [28], exploit similar overlap‐driven recombination principles, providing additional options for assembling large DNA constructs.

The flexibility and sequence independence of homology‐directed assembly make it particularly suitable for integration with other DNA manipulation strategies. By providing homologous overlaps at fragment ends, HR‐based methods can seamlessly join DNA segments generated through diverse upstream processes. This flexibility has been further enhanced through hybrid strategies (Figure 2C). For example, Nimble Cloning, which employs the rare‐cutting enzyme *Sfi* I, enables rapid plasmid linearization without extensive fragment domestication [26]. In a second hybrid approach, CRISPR‐Gibson hybrid strategies use Cas9 guided by a user‐designed single guide RNA (sgRNA) to generate targeted double‐stranded breaks at virtually any desired location in a vector. PCR‐amplified inserts with homologous overlaps can then be seamlessly integrated via Gibson assembly [84]. These approaches have been used to construct complex assemblies, including rapamycin polyketide synthase modules [85], a UAS‐cDNA/ORF library of over 5500 *Drosophila* genes conserved in humans [86], and entire biosynthetic clusters including fengycin [87]. These combinations leverage the sequence independence of HR‐based assembly with the precision of nuclease systems, significantly expanding the design space for reconstructing large biosynthetic clusters and generating high‐diversity pathway libraries.

### 2.3 Programmable nuclease‐guided assembly methods for precision and automation in pathway design

Programmable nucleases have emerged as powerful tools for in vitro DNA assembly, offering unparalleled precision and automation in constructing large DNA fragments (Figure 2D). Precision arises from programmable target recognition and cleavage: Cas12a (Cpf1) guided by crRNA recognizes TTN‐type protospacer adjacent motif (PAM) sites and generates defined 4–5 bp 5′ overhangs, enabling directional and predictable assembly of multiple fragments, as implemented in the C‐Brick system [29]. However, conventional Cpf1 cleaves variably depending on the target sequence and crRNA design, often cutting at multiple sites and producing inconsistent ends that compromise assembly accuracy. As shown in Figure 2D, the Cpf1‐assisted cutting and Taq DNA ligase‐assisted ligation (CCTL) method employs a 17‐nt crRNA to confine cleavage to defined sites, generating predictable 8‐nt sticky ends. Combined with Taq DNA ligase’s high‐temperature, high‐specificity ligation, it markedly improves the precision and efficiency of large DNA construct modification [30].

In parallel, *Pyrococcus furiosus* argonaute (PfAgo) is a programmable nuclease that does not rely on PAM sequences. Thermal denaturation of dsDNA allows strand separation, followed by site‐specific cleavage using 5′‐phosphorylated DNA guides, and reannealing to produce defined sticky‐end double‐stranded breaks [88]. PfAgo has been incorporated into the fully automated PlasmidMaker platform (Figure 2D), enabling end‐to‐end plasmid construction from primer design to ligation with high throughput and accuracy [31]. It enables high‐throughput assembly of plasmids up to 27 kb and 11 fragments, including constructs with high GC content (up to 77%) and multiple repeated sequences. These programmable nuclease strategies enable precise, predictable assembly of multiple gene modules and allow automated high‐throughput construction, directly facilitating the combinatorial design, rapid iteration, and optimization of heterologous metabolic pathways in microbial cell factories.

## 3 IN VIVO ASSEMBLY STRATEGIES FOR THE CONSTRUCTION OF HETEROLOGOUS METABOLIC PATHWAYS

In vitro assembly methods provide high flexibility and efficiency for constructing metabolic pathways; however, their efficiency decreases significantly as the number and size of DNA fragments increase [21, 89]. Large biosynthetic pathways—such as natural product BGCs, which typically span tens to hundreds of kb [37, 69, 87]—are primarily assembled in vivo through HR within host cells. In general, in vivo assembly achieves higher efficiency and greater stability than in vitro recombination methods [90], thereby accelerating the development of microbial cell factories and the optimization of biosynthetic pathways. The Gram‐negative bacterium *E*. *coli* (Figure 3A) and the Gram‐positive bacterium *S*. *cerevisiae* (Figure 3B) are among the most widely used hosts for the in vivo assembly of BGCs [38, 69, 87] and biochemical pathways [39, 40]. These assembled constructs enable two production routes: direct synthesis of the target metabolite in the original host or transfer of the plasmid to a specialized production chassis (Figure 3C). Moreover, in vivo recombination strategies have been extended to various other hosts, significantly advancing the fields of synthetic biology and metabolic engineering.

**FIGURE 3:**
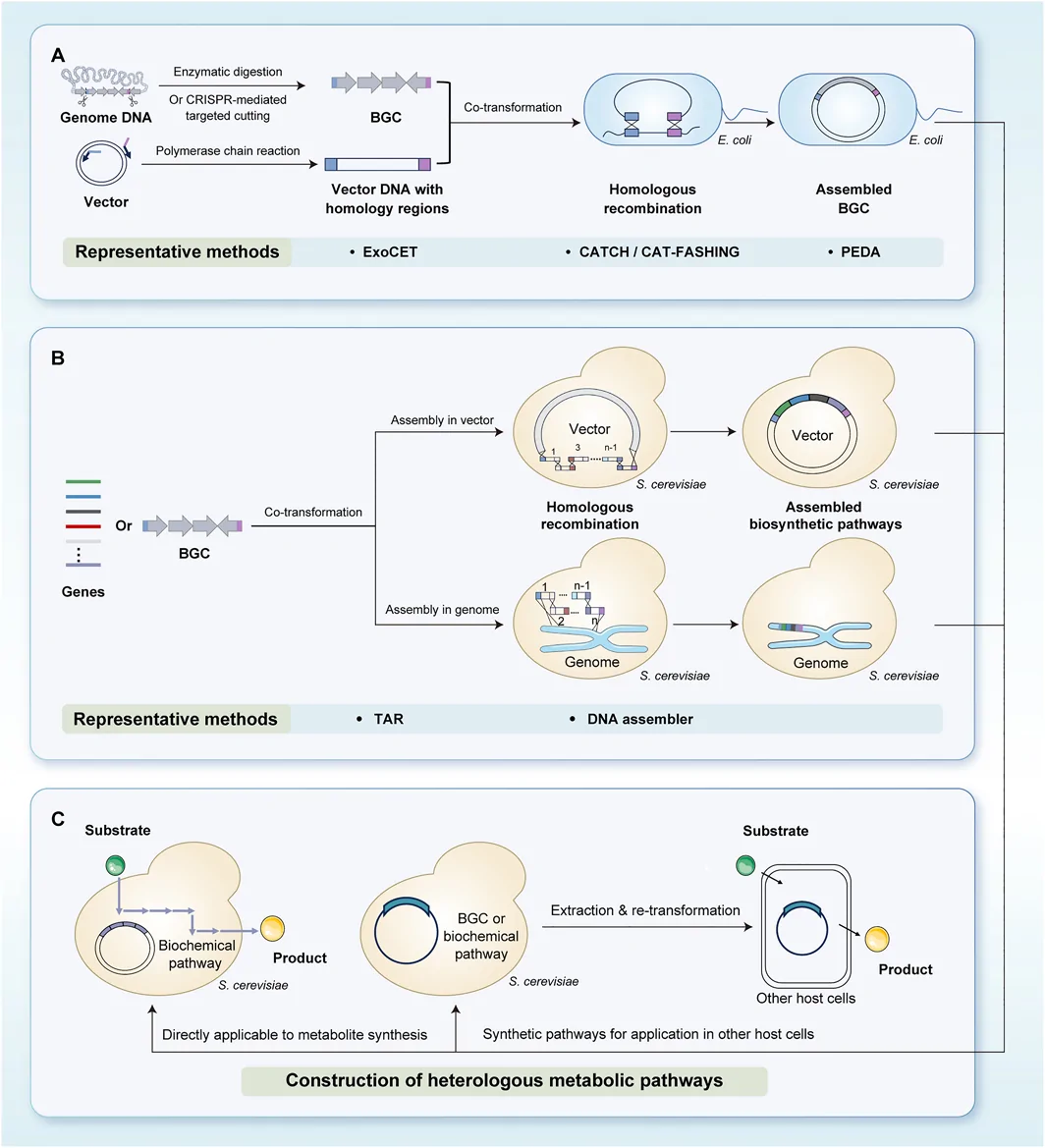
In vivo assembly strategies for the construction of heterologous metabolic pathways. In vivo DNA assembly in (A) *E. coli* and (B) *S. cerevisiae*. (C) Applications in cell factory engineering (as illustrated by the yeast system).

### 3.1 In vivo DNA assembly in *E*. *coli* for the cloning and refactoring of BGCs

*E*. *coli* is a preferred host for DNA cloning and maintenance because its endogenous recombination can be minimized more thoroughly than in any other host, making it particularly suited for assembling secondary metabolite pathways that usually contain highly repetitive DNA regions (such as polyketide BGCs). However, endogenous RecA‐mediated HR in *E*. *coli* is inefficient, and exogenous linear dsDNA introduced for assembly is degraded by the RecBCD complex, resulting in low assembly efficiency in vivo [91]. To overcome these limitations, the Red recombination system from λ phage (comprising Exo, Beta, and Gam proteins) and the RecET system from Rac phage (comprising RecE and RecT proteins) have been developed to significantly enhance HR efficiency in *E*. *coli*. Although both systems enhance recombination efficiency in *E*. *coli*, the Red system is more effective for recombination between linear and circular DNA. In contrast, RecET efficiently mediates recombination between two linear DNA molecules. Fu et al. [32] first developed the linear‐linear homologous recombination (LLHR) technique in 2012 using full‐length RecE and RecT. Using LLHR, several BGCs have been successfully assembled, including the syringolin biosynthetic gene cluster (*syl*CDE, 19 kb) from *Pseudomonas syringae* [92], the luminmycin biosynthetic pathway (18 kb) from *Photorhabdus luminescens* [68], the sevadicin biosynthetic gene cluster (11.6 kb) from *Paenibacillus larvae* [69] and polyketide secondary metabolite BGCs from *Colletotrichum higginsianum* [33]. BGCs are typically obtained through enzymatic digestion of genomic DNA, but the limitation of restriction sites may hinder the release of BGCs. Ongoing improvements (Figure 3A) have incorporated CRISPR‐mediated targeted cutting systems, such as Cas9‐Assisted Targeting of CHromosome segments (CATCH) [93, 94] and CRISPR/Cas12a‐mediated Fast Direct Biosynthetic Gene Cluster Cloning Platform (CAT‐FASHING) [95], which enable rapid and efficient capture of larger BGCs. Beyond pathway assembly, combining RecET with Red recombination or CRISPR/Cas systems has enabled effective refactoring of targeted BGCs via promoter engineering and RBS optimization, significantly enhancing the yield of natural products [96, 97]. Using the recently developed CRISETR (CRISPR/Cas9 and RecET‐mediated Refactoring) strategy, He et al. [97] refactored the 74‐kb daptomycin BGC by introducing diverse combinations of promoters with varying strengths, which resulted in a 20.4‐fold yield improvement of daptomycin.

The efficiency of direct cloning is limited by the chance that both the linear cloning vector and the target BGC simultaneously enter an *E*. *coli* cell during electroporation. In 2018, researchers developed the Exonuclease Combined with RecET recombination (ExoCET) method, which employs an initial in vitro step that exploits the 3'→5' exonuclease activity of T4 polymerase to preanneal linear vectors with genomic fragments [34]. This approach enabled the successful construction of large BGCs that had previously been challenging to clone using RecET recombination alone. Notably, whereas a large 106 kb fragment previously required cloning in three separate steps, ExoCET enables single‐step cloning, substantially improving the efficiency of BGC construction.

### 3.2 In vivo DNA assembly in *S*. *cerevisiae* for the construction of multigene metabolic pathways

*S*. *cerevisiae* exhibits a high HR rate, where DNA fragments with 40 bp of overlapping sequence achieve assembly efficiencies exceeding 90% [39, 98, 99]. Leveraging its ability to recombine small homologous and divergent DNA sequences during transformation, Larionov et al. [35] developed the transformation‐associated recombination (TAR) method in 1996, which enables selective and accurate cloning of chromosomal regions from complex genomes (Figure 3B). Their findings indicated the highest yield of yeast artificial chromosomes (YACs) when using vectors lacking autonomously replicating sequence (ARS) elements. However, for high G + C DNA constructs with a total size of over 200 kb, stable maintenance proved unattainable without integrating ARS sequences [36]. The updated TAR vector incorporates a yeast selectable marker, ARS, and a centromere sequence to ensure proper propagation, segregation, and selection in *S*. *cerevisiae*. The efficiency of TAR remains low (0.1%–2%) due to nonhomologous end joining (NHEJ), which often results in misassembly [100]. Several strategies have been developed to enhance TAR efficiency, including: (1) separating essential elements (yeast episome and selection marker) [101]; (2) introducing the *URA3* gene as a counterselectable marker [102]; (3) using the α subunit of K toxin as a counterselectable marker [37]; and (4) employing CRISPR/Cas9 to pretreat genomic DNA, which increased region‐positive cloning efficiency by 35‐fold [103]. Recently, to overcome the challenge of low efficiency in capturing BGCs and difficulty in stably maintaining large BGCs (e.g., glycopeptide antibiotic BGCs, typically >70 kb) using traditional TAR methods, Xu et al. [38] developed an optimized system by optimizing the expression of counter selection marker *URA3*, reasonably designing hook sequences, and incorporating a copy‐number control replicon (oriV‐ori2‐repE‐sopABC). This optimized system achieved a 70% capture efficiency for the 76‐kb corbomycin BGC, a substantial improvement over the traditional TAR efficiency (below 10%). Notably, the captured BGCs demonstrated stable integration and efficient expression in other hosts, which achieved a 19‐fold increase in the final corbomycin yield on the GPAHex platform. TAR eliminates the need for in vitro manipulation and enables the assembly of extremely long DNA fragments, pioneering HR‐mediated assembly in yeast cells. This method has been widely applied to assemble individual or entire genomes [104, 105, 106, 107], assemble BGCs from complex genomes [37, 108, 109], and reconstruct gene clusters for synthetic biology applications [110, 111, 112]. More recently, TAR has been integrated into an automated biofoundry platform, significantly improving the scalability and precision of large‐scale DNA assembly [113]. This platform enables the construction of expression‐tunable multigene libraries, such as the β‐carotene metabolic pathway.

Similarly, Shao et al. [39] developed the DNA assembler method in *S*. *cerevisiae* in 2009, enabling the one‐step assembly of multiple gene expression cassettes into biosynthetic pathways via in vivo HR. Functional biosynthetic pathways ranging from 9 to 19 kb—including a three‐gene D‐xylose utilization pathway, a five‐gene zeaxanthin biosynthetic pathway, and a combined eight‐gene D‐xylose utilization and zeaxanthin biosynthetic pathway—can be rapidly assembled on either plasmids or chromosomes [39, 40]. Beyond pathway assembly, the DNA assembler has also been employed to optimize metabolic pathways using customized optimization of metabolic pathways by combinatorial transcriptional engineering (COMPACTER), enabling rapid tuning of gene expression in xylose and cellobiose utilization pathways [114]. Currently, DNA assembler‐assembled plasmids are applied in targeted genome editing [115], metabolite synthesis [116], and unconventional carbon source utilization [117].

### 3.3 In vivo DNA assembly in other microorganisms

Advances in sequencing and HR technologies have facilitated the adaptation of in vivo DNA assembly for use in a diverse range of microorganisms. *Bacillus subtilis* (*B*. *subtilis*) has been one of the most widely used hosts for DNA assembly. Owing to its natural high extracellular DNA transformability, extracellular ssDNA can readily enter the cytoplasm of *B*. *subtilis* and integrate into the genome via RecA‐mediated HR. Several in vivo assembly methods have been developed in *B*. *subtilis*, including inchworm elongation (IWe), domino assembly, and inducible RecA expression BGM vector (iREX) [118, 119, 120]. Comprehensive reviews of DNA assembly techniques in *B*. *subtilis* have been provided by Li et al. [121]. Beyond *E*. *coli*, *S*. *cerevisiae*, and *B*. *subtilis*, in vivo DNA assembly methods have also been established in various other microorganisms [42, 43, 44, 122]. In NHEJ‐deficient strains of *Aspergillus nidulans*, efficient DNA fragment fusion requires only 25 bp sequence overlaps, facilitating the construction and optimization of metabolic pathways [42]. Shi et al. developed an efficient CRISPR/Cas9‐based genome editing and one‐step DNA assembly system in *Starmerella bombicola*, enabling the efficient assembly of multiple DNA fragments for the production of single acid‐type sophorolipids with high yield and low cost [43]. More recently, a method termed simultaneous in vivo assembly and targeted genome integration of multiple DNA fragments (SATIMD) was developed in *Trichoderma reesei* (*T*. *reesei*). SATIMD employs CRISPR/Cas9 to introduce specific DSBs in the genome, thereby enhancing HR efficiency and enabling the assembly of a 32.7‐kb sorbicillinoid BGC via the one‐step integration of 10 DNA fragments at a specific locus [44]. This suggests that SATIMD is a powerful tool for heterologous gene cluster assembly, thus speeding up cell factory construction. Notably, neither the number of fragments nor the length of flanking homology arms affected assembly efficiency in *T*. *reesei*, a finding that warrants further investigation.

Direct assembly of large DNA fragments in hosts with limited HR capacity remains challenging. Increasing HR efficiency within these hosts offers the most direct and effective solution. Although recombination systems such as Red and RecET—developed initially in *E*. *coli*—have been widely used to improve HR efficiency in many Gram‐negative bacteria, their application is often restricted to specific species [123]. To overcome this limitation, Huang et al. [122] identified an operon (XBJ1_operon 0213) from *Xenorhabdus bovienii* that encodes RecET‐like recombinases. Expression of this operon significantly enhanced recombineering efficiency in both *E*. *coli* and *X*. *stockiae*, enabling the development of a robust recombineering system in *X*. *stockiae*. This technology holds significant application value in the identification and modification of BGCs in natural products. In a separate study, researchers developed a phage enzyme‐assisted in vivo DNA assembly (PEDA) method [124] by combinatorially expressing T5 exonuclease and T4 DNA ligase (Figure 3). In this system, T5 exonuclease generates single‐stranded overhangs from linear DNA ends, whereas T4 DNA ligase seals nicks after annealing of homologous sequences. Remarkably, PEDA enables the assembly of fragments with homologous overlaps as short as 5 bp in *E*. *coli* and is applicable to a broad range of species, including *Ralstonia eutropha*, *Pseudomonas putida (P. putida)*, *Lactobacillus plantarum*, and *Yarrowia lipolytica*. Using this method, a plasmid library containing different RBS variants was constructed through direct in vivo DNA assembly in *P*. *putida* KT2440, confirming the applicability of PEDA for metabolic flux regulation.

## 4 SYNTHETIC GENOME ASSEMBLY ENABLES GENOME‐SCALE REPROGRAMMING OF HOST METABOLISM

The primary significance of synthetic genome assembly lies in its capacity for host metabolic reprogramming at the genome scale (Figure 4). As an extension of in vivo assembly, it uses the wild‐type genome as a reference, employing a hierarchical strategy to construct synthetic segments and stepwise replace native genomic sequences, driving key innovations in large DNA fragment assembly [48, 55, 58]. Now, advances in synthetic genome assembly have progressed from viral genomes to prokaryotic genomes and then to multiple chromosomes in eukaryotic genomes [125, 126, 127]. De novo genome synthesis facilitates genome‐wide engineering, endowing synthetic strains with expanded or enhanced industrial capabilities, such as global codon recoding for noncanonical amino acid (ncAA) incorporation [128, 129], antiviral immunity [130, 131], and insertion of global flexibility elements for accelerated evolution [132].

**FIGURE 4:**
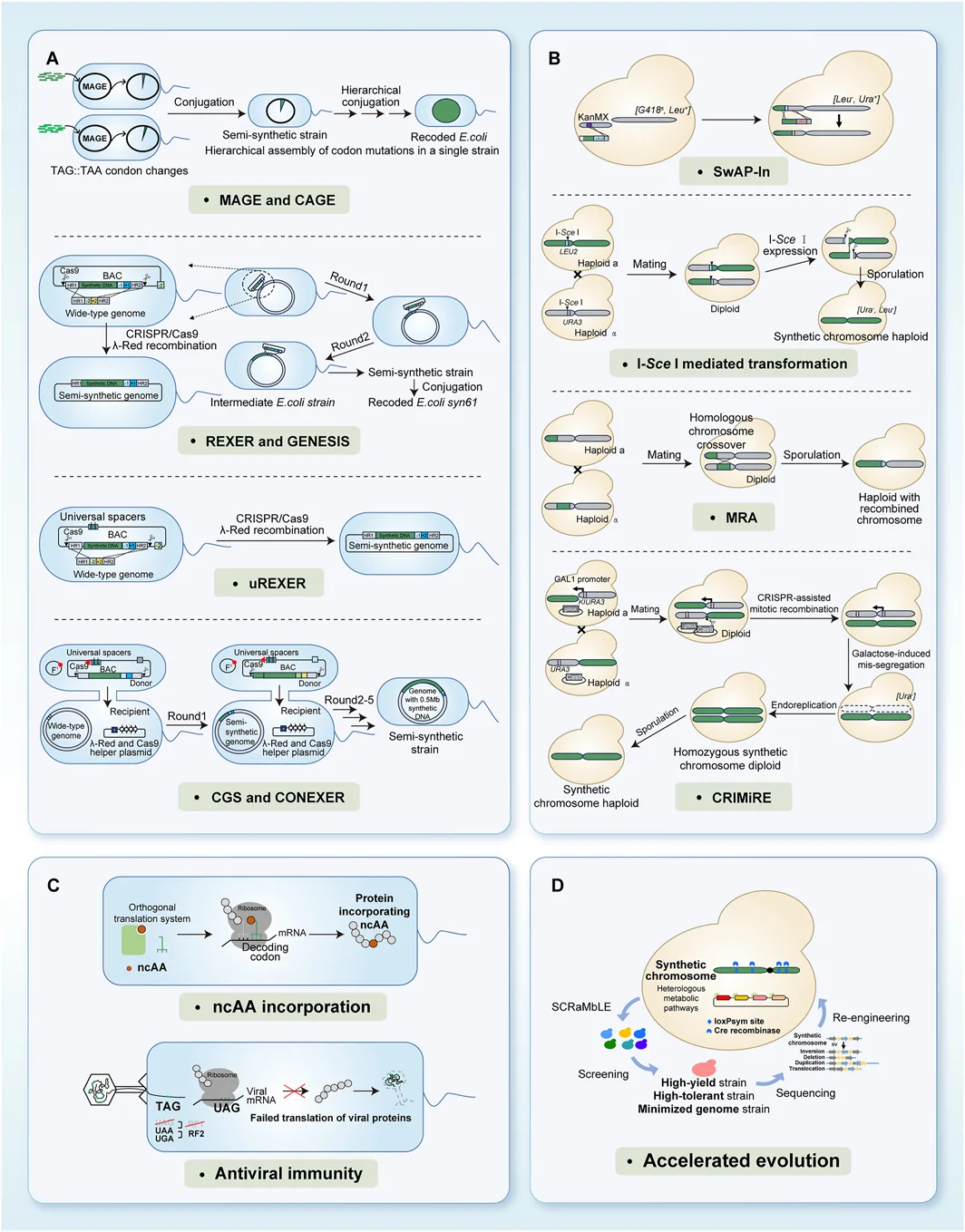
Synthetic genome assembly technologies drive applications of genomically recoded *E. coli* and synthetic yeast. The representative synthetic genome assembly methods in (A) *E*. *coli* and (B) *S. cerevisiae*. (C) Genome‐wide codon removal in *E*. *coli* facilitates application of ncAA incorporation and antiviral immunity. (D) Genome‐wide loxPsym insertion in a synthetic yeast chromosome allows accelerated evolution. The synthetic chromosomal fragments are represented in green, and the wild‐type chromosomal fragments are represented in gray.

### 4.1 Development of de novo synthetic genome assembly technologies

Synthetic genome assembly technology has evolved from viral to prokaryotic and then to eukaryotic genomes, leading to significant enhancements in length, accuracy, speed, and stability of genome assembly. For smaller viral genomes, the complete synthetic genomes of poliovirus (about 7 kb) [125] and ΦX174 bacteriophage (6 kb) [133] could be assembled in vitro with polymerase cycling assembly (PCA)‐derived methods. For large genomic constructs ranging from 10 kb to Mb, where DNA fragments exhibit instability, the yeast‐based TAR approach is employed. This method facilitates the combination of small DNA fragments with a YAC vector to form genome‐YAC hybrids in yeast cells, enabling the assembly of large virus genomes such as SARS‐CoV‐2 [134] and mycoplasma genomes such as *Mycoplasma genitalium* (583 kb) [126] and *Mycoplasma mycoides* (1.08 Mb) [135].

For bacterial genome synthesis at the Mb scale, stepwise assembly of large synthetic fragments (>100 kb) is the principal strategy. Synthetic genome assembly enables the genome‐wide removal of target codons, which can be reassigned for producing novel materials. To assemble a recoded *E*. *coli* genome with complete elimination of UAG stop codons, Isaacs et al. [7] used Multiplex automated genome engineering (MAGE) to modify 314 TAG‐containing genes in the genome and created conjugative assembly genome engineering for codon mutation fragment transfer and assembly (Figure 4A). However, MAGE introduces off‐target mutations and is unsuitable for removing the much more abundant sense codons [7, 136]. To further compress to 61 codons, the replicon excision enhanced recombination (REXER), a dual‐selection marker genome integration technique, was used [45]. REXER utilizes CRISPR/Cas9 and λ‐Red recombination systems to drive efficient and precise replacement of wild‐type genomic segments (Figure 4A). The iterative marker‐swapping process, termed genome stepwise interchange synthesis (GENESIS), combined with conjugation, enabled the construction of the final synthetic genome strain Syn61 [137]. To deeply compress the genetic code, George Church’s team worked toward constructing a fully 57‐codon recoded synthetic strain (*rE*.*coli*‐57 or *Ec*_Syn57). Their strategy employed the yeast TAR method to assemble about 50 kb of recoded fragments for stepwise genome replacement [138] and a multiomics‐based iterative construction and troubleshooting (SynOMICS) approach to identify and correct issues in the synthetic genome assembly [139]. In contrast, Jason Chin’s team developed the uREXER method based on REXER, which employs universal CRISPR/Cas9 spacers to streamline the process (Figure 4A). Through iterative uREXER (GENESIS assembly) and conjugation assembly, these recoded fragments were compiled into a single chassis, Syn57 [46]. Moreover, the team also upgraded the continuous genome synthesis method—conjugation coupled with programmed excision for enhanced recombination (CONEXER). Compared to REXER, CONEXER integrates bacterial conjugation and omits intermediate sequencing (Figure 4A), facilitating single integration from 4 days to 1 day [47].

In eukaryotic genome synthesis, Sc2.0 is a milestone project aimed at the complete synthesis of yeast chromosomes. A core principle involves genome‐wide introduction of inducible genetic flexibility elements to enable genome rearrangement for phenotypic evolution of yeast strains [48]. To achieve these fully synthetic yeast chromosomes, the standardized switching auxotrophies progressively for integration (SwAP‐In) method was developed [127] (Figure 4B). This method progressively replaced native chromosomal regions with synthetic megachunks (30–50 kb) by employing alternating selection with auxotrophic markers (*URA3* and *LEU2*), successfully generating several synthetic chromosomes, including synI [49], synIII [127], synV [50], synVI [51], synIX [52], synX [53], and synXIV [54]. Although the SwAP‐In method minimizes off‐target effects by utilizing yeast HR, multiple rounds of replacement make the assembly process time‐consuming, and efficiency decreases as the number of rounds extends. To accelerate this process, I‐*Sce* I‐mediated transformation enabled parallel replacement from both chromosome ends in haploids, followed by diploid hybridization and cleavage by I‐*Sce* I to successfully assemble chromosomes synII [55], synVII [56], and synXIII [57] (Figure 4B). This method reduces the total assembly time by almost 50%, significantly boosting synthetic chromosome assembly efficiency. The meiotic recombination‐mediated assembly method further streamlines and accelerates the assembly process by combining segments from different haploid strains through meiosis (Figure 4B), ultimately assembling chromosomes synIV [59], synVIII [60], synXII [58], and synXVI [61]. However, the recombination process during assembly is random, and cross‐mixing with wild‐type sequences becomes a major bottleneck, especially for long synthetic chromosomes. CRISPR/Cas9‐mediated mitotic recombination with endoreduplication (CRIMiRE) leverages the unique cleavage sites of CRISPR/Cas9 to ensure chromosome recombination at precise locations and generates a homozygous diploid configuration to prevent cross‐mixing with wild‐type sequences, enabling efficient assembly of synXI [62] and synXV [63] (Figure 4B). After the assembly of individual synthetic chromosomes, multichromosome consolidation strategies such as “endoreduplication intercross” and “KAR1 mutation” have successfully combined up to 6.5 synthetic chromosomes in a single strain, bringing the goal of a fully synthetic yeast genome within the immediate future [140, 141].

### 4.2 Applications of synthetic genome‐engineered *E*. *coli* in ncAA incorporation and antiviral immunity

The assembly of the *E*. *coli* synthetic genome has created genomically recoded organisms, such as synthetic *E*. *coli* with stop codons swapped from TAG to TAA, Syn61, *rE*.*coli*‐57 (or *Ec*_Syn57), and Syn57, which free up 1–7 codons. These strains accelerate genetic code expansion (GCE) technology development by employing orthogonal translation systems (aminoacyl‐tRNA synthetase/tRNA pairs). By harnessing this system, vacant codons can be reassigned to incorporate ncAAs into target proteins, producing novel peptide drugs, biopolymers, and industrial enzymes (Figure 4C). For instance, the *C321*.Δ*A* *E*. *coli* strain, featuring genome‐wide UAG to UAA codon replacement and RF1 knockout, converts UAG into a functional sense codon for ncAA insertion. Lajoie et al. [142] employed this system to achieve high efficiency and specific insertion of pAcF with undetectable readthrough. Cheng et al. [143] achieved efficient Sec incorporation at UAG codons, enabling production of mammalian selenoprotein thioredoxin reductases with unsurpassed purity and yield. To improve the yield of ncAA‐containing proteins, Yi et al. [144] integrated the T7 RNA polymerase system into *C321*.Δ*A*, achieving a 2.5‐fold increase in target protein production. Furthermore, Grome et al. [145] compressed stop codons into a single codon, generating the *C321*.Δ*A ΔTGA* variant. Both UAG and UGA codons were reassigned to enable multisite incorporation of two ncAAs into a protein with >99% accuracy. In the Syn61 strain, genes decoding vacant codons were deleted (Syn61Δ3), and three mutually orthogonal ncAA incorporation systems were introduced. By reassigning TCG, TCA, and TAG codons, Robertson et al. achieved simultaneous incorporation of three ncAAs into proteins, producing diverse noncanonical heteropolymers and macrocycles [128]. Spinck et al. utilized the Syn61Δ3 platform to encode five distinct ncAAs (AllocK, AlkynK, pIF, CbzK, and pAzF) at TCG and TAG codons in the macrocycle‐encoding genes, generating 25 macrocyclic peptides, each incorporating two distinct ncAAs [129]. Through targeted hydroxy acid incorporation, they also achieved the synthesis of 12 diverse non‐natural depsipeptide macrocycles. With the synthesis of Syn57, which has created a deeply recoded strain to genetically encode up to seven distinct ncAAs into proteins, and the synthesis of noncanonical macrocycles and polymers comprising up to seven novel monomers [46]. These achievements demonstrate the immense potential of synthetic genome‐assembled strains for functional protein biomanufacturing.

On the other hand, recoding the *E*. *coli* genome to reassign the UAG stop codon effectively creates a virus‐resistant host, providing high biosafety for industrial fermentation. As shown in Figure 4C, in the UAG codon‐ and RF1‐deficient host C321.ΔA, it disrupts proper termination of T7 phage (genome contains 6 UAG among 60 stop codons) gene expression, preventing viral amplification and propagation [142]. Ma et al. also discovered that the C321.ΔA strain exhibits multiviral (λ, M13, P1, and MS2) resistance at titers up to 10^11^ PFU/mL and impaired conjugative plasmids (F and RK2) up to 10^5^‐fold [146]. The Syn61Δ3 strain, with more deletions of codons and translation components, exhibits broad‐spectrum phage resistance, with negligible growth impact from the phage cocktail (λ, P1vir, T4, T6, and T7) [128]. In addition, the Syn61Δ3 strain, with its refactored genetic codes, not only resists viruses written in the canonical code but also blocks genetic information leakage from synthetic organisms to natural organisms [130]. These synthetic genome‐assembled strains provide a genetic firewall against the natural ecosystem; they thus represent a secure production chassis for pharmaceuticals and bioactive compounds.

### 4.3 Applications of the synthetic chromosome yeast for accelerated evolution

In the Sc2.0 project, to introduce inducible genetic flexibility into synthetic chromosomes, thousands of symmetric Cre‐loxP recombination (loxPsym) sites were inserted into the 3′ UTR of each nonessential gene, enabling a Cre recombinase‐mediated rearrangement of synthetic chromosomes called synthetic chromosome rearrangement and modification by loxP‐mediated evolution (SCRaMbLE). These modifications, including deletions, inversions, duplications, and translocations of chromosome regions, generate combinatorial stochastic diversity of synthetic chromosomes [147]. This has been shown to accelerate genome‐wide metabolic regulation and evolution in yeast, enabling applications in conditional tolerant strain engineering, natural compound production enhancement, and chassis cell simplification (Figure 4D). In terms of enhancing strain tolerance under environmental stress conditions, Luo et al. [148] used SCRaMbLE and efficient selection based on a loxP‐mediated switch of two auxotrophic markers (ReSCuES) in the synXII strain, generating three yeast strains capable of growing at 39.5°C, improving the OD600 value by 21‐fold under 0.6% acetic acid conditions, and generating a tolerant strain capable of growing in 8% ethanol. Ma et al. [149] employed SCRaMbLE in yeast strains harboring either one (synV) or two (synV and synX) synthetic chromosomes, obtaining alkali‐tolerant strains capable of withstanding pH 8.0. Whole‐genome sequencing analysis confirmed that the deletion of *SPT2* was responsible for the enhanced alkali tolerance. Additionally, researchers also screened strains with improved tolerance to antibiotics such as rapamycin [150] and hygromycin [151], as well as growth‐affecting factors such as caffeine [152] and high salt [153] via SCRaMbLE.

In terms of sugar utilization, Blount et al. [154] introduced the xylose metabolic pathway into the synV strain. By SCRaMbLE, they successfully obtained a strain with a 5.85‐fold improvement in growth capability on a medium where xylose was the sole carbon source. Structure variant analysis revealed that the deletion of *MXR1* was critical for xylose utilization, offering potential for environmentally friendly application of yeast. In enhancing the production of natural compounds, Zhang et al. [155] achieved a 15.8‐fold increase in lycopene yield by applying SCRaMbLE in a diploid strain harboring synII, attributing part of this improvement to the deletion of the *PEX32* gene. Jia et al. [156] employed a combinatorial approach using an AND gate to enable precise control of SCRaMbLE and multiplex SCRaMbLE iterative cycling (MuSIC) in a diploid yeast carrying synIII and synV. After five cycles, they achieved a 38.8‐fold increase in carotenoid production. Additionally, the SCRaMbLE of synthetic chromosome yeast has demonstrated potential in boosting the biosynthesis of more compounds, including violacein [157], astaxanthin [158], betulinic acid [159], and geraniol [160], as well as phenylethyl acetate and phenylethylamine [161]. Furthermore, SCRaMbLE‐based systems can also be employed to build metabolically simplified chassis cells by deleting nonessential chromosomal genes. This strategic reduction will create a cleaner background for heterologous gene expression, helping improve metabolic resource partitioning and enhance metabolic pathway efficiency [162, 163].

## 5 SYNTHESIZING NeoChrs FOR EXPANDING COMPLEX BIOSYNTHETIC NETWORKS

Constructing efficient *S*. *cerevisiae* cell factories typically requires the integration of multiple exogenous genes into native chromosomes. For example, the implementation of the complete QS‐21 biosynthetic pathway necessitated the introduction of 38 heterologous genes from seven enzyme families [164]. However, integrating such large genetic constructs might disrupt chromosomal architecture, potentially affecting the expression of neighboring genes and compromising chromosome replication. Given that *S*. *cerevisiae* exhibits tolerance to variations in chromosome number [165, 166], synthetic NeoChrs—which are modular, supernumerary artificial chromosomes providing an orthogonal platform for stable, high‐capacity endogenous or/and heterologous gene expression without disrupting native chromosomal function—have emerged as platforms for reprogramming native cellular processes and introducing novel functionalities [5, 64, 66]. In contrast to the construction of synthetic chromosomes in Sc2.0, which relies on existing genomic frameworks [48, 55], there are no native templates for the design of NeoChrs, and thus, NeoChrs need to be de novo designed from individual accessory genes and regulatory elements. To assemble NeoChrs, structural elements (centromere, ARS, telomeres, and auxotrophic markers), along with PCR‐amplified essential gene sequences, were cotransformed into *S*. *cerevisiae* for in vivo assembly. The overall construction workflow of NeoChr is illustrated in Figure 5A.

**FIGURE 5:**
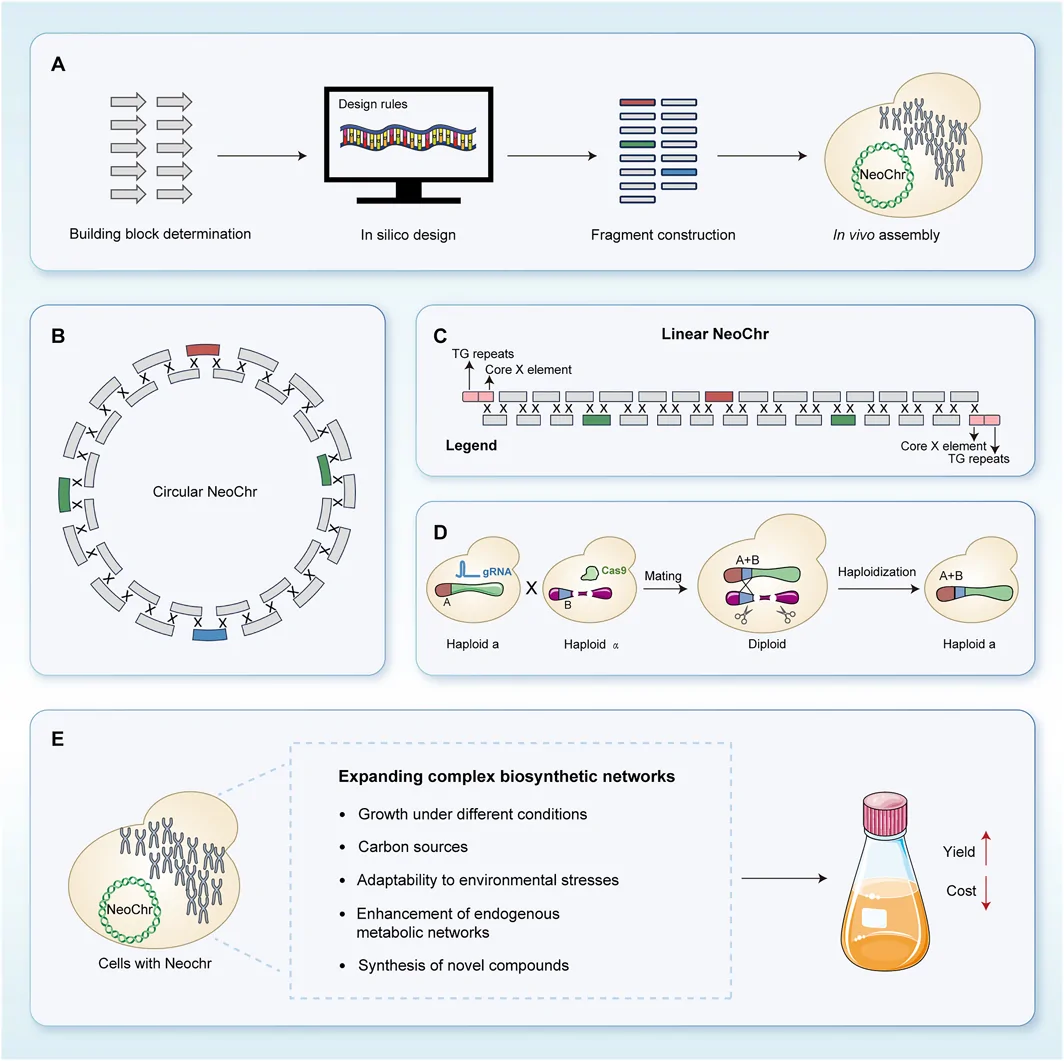
De novo construction of NeoChrs. (A) Schematic representation of the design and construction of NeoChrs. The in vivo assembly design for (B) circular NeoChrs and (C) linear NeoChrs. (D) Schematic illustration of CRISPR/Cas9‐mediated haploidization. The red boxes represent the centromere for segregation. The green boxes represent ARS for replication. The blue boxes represent the telomerator for circular NeoChr. The pink box represents Tess for linear NeoChr. (E) NeoChr expands the capabilities of microbial cell factories (as illustrated by the yeast system). ARS, autonomously replicating sequence.

### 5.1 Constructing NeoChrs via de novo design and assembly

Several synthetic NeoChrs have been constructed to expand the capabilities of microbial cell factories. In 2021, Postma et al. [64] exploited yeast HR to assemble up to 44 DNA parts in a single step into modular circular NeoChrs (Figure 5B). Specifically, they first assembled TUs—each consisting of a promoter, gene, and terminator—via Golden Gate cloning. Subsequently, they de novo assembled these fragments, which featured 60 bp nongenomic overhangs, via the highly efficient HR machinery of *S*. *cerevisiae*. The stability of NeoChrs and their effect on host physiology are key considerations for their application in synthetic biology. Interestingly, the introduction of four differently sized empty NeoChrs resulted in reduced specific growth rates and the loss of NeoChrs in approximately 20% of the population [64]. To address this, the authors hypothesized that a linear configuration mimicking native yeast chromosomes could improve performance. They constructed a 100‐kb linear NeoChr using short telomere seed sequences for telomere formation (Figure 5C) [5]. However, results showed that linear NeoChrs exhibited lower assembly efficiency and fidelity compared to their circular counterparts, although both types were comparable in growth rate, stability, and phenotypic effects.

Because of the limitations of one‐step assembly—namely, (1) the reduced efficiency of in vivo assembly as the number of fragments increases, and (2) the decreased probability that all transformed fragments will successfully enter the nucleus—large‐size NeoChrs are typically constructed through iterative genome assembly involving multiple rounds of HR with a small number of fragments. Mitchell et al. developed the eSwAP‐In strategy (extrachromosomal switching auxotrophies progressively for integration) [167]. This method relies on iterative use of two different auxotrophic markers (*URA3* and *LEU2*) embedded near the rightmost ends of DNA fragments that are designed for stepwise assembly. Applying the eSwAP‐In strategy, Kutyna et al. [66] designed and constructed a NeoChr incorporating diverse elements from the *S*. *cerevisiae* pan‐genome, enabling the Sc2.0 parent strain to acquire a wide range of traits found in industrial and environmental isolates. Specifically, 17 unique genomic segments were identified from over 200 *S*. *cerevisiae* strains and integrated in silico into a single 211 kb DNA molecule, termed the pan‐genomic NeoChr (PGNC). The design followed Sc2.0 principles, including substitution of all TAA stop codons with TAG, insertion of watermarks in 36 ORFs, and placement of 63 loxPsym sites. The authors compared circular (PGNC^circ^) and linear (PGNC^lin^) chromosomal variants, observing that the growth curve of PGNC^circ^ was comparable to that of the wild‐type strain, whereas PGNC^lin^ showed a slightly extended lag phase and reduced cell density and maximum specific growth rate [66]. These findings imply that intracellular mechanisms governing NeoChr stability and function remain to be fully understood. Future improvements may rely on screening additional combinations of structural and regulatory elements.

The iterative processes involve the extraction of the assembled DNA from yeast, enrichment in vitro or in *E*. *coli*, and retransformation into yeast for subsequent rounds of assembly. To improve the import and export efficiency of large DNA across host cells and thereby increase overall assembly efficiency, He et al. developed a yeast life cycle‐mediated iterative assembly method (YLC‐assembly) [168]. In this approach, mating between haploid yeast strains brings two DNA fragments into a diploid cell for recombination, after which meiosis generates spores that can be used in the next assembly cycle. Although YLC‐assembly achieves a high success rate for megabase‐scale DNA assembly, each round takes approximately 11 days due to the sporulation process required [168]. To overcome this limitation, a haploidization‐based DNA assembly and delivery strategy in yeast (HAnDy) was recently developed, enabling the de novo assembly of a 1.024‐Mb NeoChr containing 542 exogenous genes [6]. Three key design principles guided the construction of the NeoChr: (1) clustering of functionally related genes identified from 1011 *S*. *cerevisiae* isolates that are variable across the population but absent in S288C; (2) establishment of an orthogonal rearrangement system utilizing *loxP* and *vox* sites; and (3) incorporation of a precise genome editing system using “GGCC” sequences as specific gRNA target sites. The HAnDy method embeds the DNA assembly process within a CRISPR/Cas9‐mediated programmed haploidization cycle (Figure 5D). Briefly, two sets of episomal DNAs, preconstructed in haploid strains of opposite mating types, are brought together through mating. The DNAs are then assembled into a NeoChr via HR, and haploidization is completed through targeted genome elimination using CRISPR/Cas9. The resulting haploid strain can subsequently mate with another to initiate the next round of assembly.

### 5.2 NeoChrs confer phenotypic plasticity to the host strain

The widely used laboratory strain *S*. *cerevisiae* S288C—and its derivatives typically used for yeast cell factory construction—lack many genes that contribute to the phenotypic diversity observed in industrial and environmental isolates. De novo‐synthesized NeoChr function as mobile genetic modules that enhance host adaptation to various environmental stresses and enable the utilization of diverse carbon sources [6, 66]. For instance, the introduction of a PGNC into an Sc2.0‐derived strain conferred distinct phenotypes compared to the parental strain. The PGNC strain exhibited at least a 2‐fold increase in relative growth under nine different conditions, including D‐melibiose, palatinose, butyric acid, 5% sodium formate, 4% sodium lactate, neomycin, carbonyl cyanide 4‐(trifluoromethoxy) phenylhydrazone (FCCP), deoxy‐D‐glucose, and ibuprofen [66]. Notably, only the PGNC‐containing strain could metabolize alternative carbon sources such as palatinose (a disaccharide of glucose and fructose), melibiose (a disaccharide of glucose and galactose), and butyric acid. The ORF(s) responsible for these phenotypes can potentially be identified by analyzing intermediate PGNC strains obtained during stepwise assembly; consequently, their direct introduction into chassis strains can enhance tolerance to complex fermentation environments and increase product yield. Likewise, the introduction of NeoChrs assembled via HAnDy into six phylogenetically distinct yeast strains significantly improved host adaptability to various environmental stresses, including temperature, osmotic pressure, heavy metals, and the ability to use glutaric acid as a sole carbon source [6]. The NeoChr equips microbial cell factories with enhanced robustness for complex fermentation processes by enabling adaptation to environmental stresses, and the utilization of novel carbon sources further creates economic advantages by permitting the use of cheaper, renewable feedstocks (Figure 5E).

### 5.3 NeoChrs expand the metabolic network of the host strain

NeoChrs not only enhance global metabolite production but also enable the synthesis of novel compounds in *S*. *cerevisiae*. Quantitative metabolite analysis of the host strain after the introduction of HAnDy‐assembled NeoChrs confirmed the enrichment of eight endogenous metabolites, including pantothenic acid, myristic acid, and pipecolic acid. Furthermore, nine putative novel metabolites and the aromatic compound methyl anthranilate were detected [6]. Enhancing the production of key endogenous metabolites provides cell factories with abundant precursors, thereby increasing their overall biosynthetic capacity. Furthermore, enabling the synthesis of novel compounds allows for the development of new microbial production platforms and opens avenues for identifying previously unknown biosynthetic pathways (Figure 5E). In addition, NeoChrs offer significant advantages in pathway swapping [5, 64] and microbial cell factory construction [5]. The concept of modular pathway swapping involves the genetic reduction and clustering of genes from a specific metabolic pathway, allowing facile replacement with alternative configurations. This approach is particularly valuable for the metabolic reprogramming of industrial cell factories. For instance, genes encoding glycolysis have been relocated either to a single chromosomal locus [169] or to a defined landing pad on NeoChrs [5, 64]. Postma et al. [5] combined pathway swapping with NeoChrs to rewire endogenous metabolic networks and introduce new heterologous pathways in *S*. *cerevisiae*. Specifically, 20 native genes involved in glycolysis, ethanol fermentation, and the pentose phosphate pathway were relocated from native chromosomes to NeoChrs. Additionally, the whole pathway for aromatic amino acid biosynthesis—including feedback‐resistant alleles (10 genes)—along with 11 plant genes required for pelargonidin 3‐O‐glucoside (P3G) synthesis from tyrosine and phenylalanine, was assembled onto a single NeoChr. Expression of the de novo anthocyanin pathway from this NeoChr enabled anthocyanin production, whereas increasing the copy number of *coAtCHS3* and repairing *coAtANS* further enhanced biosynthetic efficiency.

## 6 SUMMARY AND OUTLOOK

This review illustrates how large DNA fragment assembly has revolutionized the construction, modification, and evolution of microbial cell factories. In vitro assembly allows flexible assembly of standardized parts or synthetic pathways without host limitation, whereas in vivo assembly offers higher efficiency and stability for larger biosynthetic pathways (>10 kb). Together, they significantly accelerate strain construction and optimization. Synthetic genome assembly elevates microbial engineering beyond local optimization to constructing purpose‐built ideal chassis. Assembly of additional NeoChrs further provides cell factories with a flexible and scalable platform, thereby enabling versatile network expansion. Ultimately, these large DNA fragment assembly techniques lay a solid foundation for industrial applications of advanced microbial cell factories in biomanufacturing.

However, large DNA fragment assembly strategies are usually applied to model organisms such as *E*. *coli* and *S*. *cerevisiae*, and their limited host diversity restricts broader application in metabolic engineering. On the one hand, future efforts should focus on developing efficient in vivo assembly systems for more hosts (e.g., *T*. *reesei*, *Vibrio natriegens*, and *Y*. *lipolytica*), expanding applications of these species in metabolic engineering and synthetic biology [44, 170, 171]. On the other hand, delivery technologies are needed to transfer assembled large DNA fragments across cells. Current methods, including bacterial conjugation [137], yeast mating [6], and induced cell fusion [172], have enabled large DNA fragment transfer between *E*. *coli* strains, yeast strains, and even across *E*. *coli* and yeast. Nevertheless, interspecies transfer efficiency remains low. Future work should therefore prioritize optimizing transfer conditions and elucidating the underlying fusion mechanisms to improve efficiency. Additionally, developing novel delivery systems to expand the host range, as well as leveraging nuclear isolation‐transplantation technologies such as synNICE for precise large DNA fragment transfer [173], are critical.

The engineering of microbial cell factories continues to be constrained by the time‐consuming and costly process of large DNA fragment assembly. Several promising avenues are emerging to address these challenges. Advances in high‐throughput DNA synthesis technologies such as microchip‐based massive parallel synthesis are poised to provide higher‐quality starting materials at a significantly reduced cost [10]. Automated high‐throughput platforms, integrated with established methods such as Biopart Assembly Standard for Idempotent Cloning (BASIC) [174], Robot‐assisted MoClo (RoboMoClo) [24], and Uracil‐Specific Excision Reagent (USER) [175], enable robust and parallel construction of complex genetic constructs. Looking forward, the integration of these modular, automated DNA synthesis and sequencing technologies with automated assembly platforms is paving the way for fully automated biological system engineering. When further combined with AI‐driven design, large DNA fragment assembly will power a high‐throughput, high‐precision Design‐Build‐Test‐Learn (DBTL) cycle. Together, these advances are expected to slash the cost and time by orders of magnitude, thereby dramatically accelerating the development cycle of microbial cell factories.

In addition, despite advances in NeoChr and genome assembly, their application in engineering microbial cell factories remains nascent. Looking ahead, NeoChr assembly supports large functional modules “plug and play.” Combining the heterologous pathway module with interconnected metabolic modules (e.g., glycolysis, precursor synthesis, and cofactor regeneration) on NeoChrs allows for facile manipulation and testing of ultralarge metabolic pathways. The NeoChr or synthetic genome assembly also enables building pan‐genomes or hybrid genomes to aggregate superior traits across hundreds or thousands of strains for expanding adaptability to complex industrial conditions [6, 66]. Moreover, the utilization of genome‐recoded chassis (e.g., GCE in *E*. *coli*) provides for secure and efficient production of novel biochemicals. This paradigm is now extending to yeast for more precise strain regulation and biocontainment [176, 177]. In the near term, we are likely to see these technologies being applied for systematic, accurate, and in‐depth engineering of industrial strains with hyper tolerance and productivity.

## AUTHOR CONTRIBUTIONS

**Yu Zhang**: Conceptualization; investigation; project administration; visualization; writing—original draft; writing—review and editing. **Wenqian Liu**: Conceptualization; investigation; visualization; writing—original draft; writing—review and editing. **Dongyuan Cheng**: Conceptualization; investigation; visualization; writing—original draft; writing—review and editing. **Jiazhang Lian**: Supervision; writing—review and editing. **Xian Fu**: Conceptualization; Funding acquisition; supervision; writing—review and editing. **Yue Shen**: Conceptualization; Funding acquisition; supervision; writing—review and editing.

## CONFLICT OF INTEREST STATEMENT

The authors declare no conflicts of interest.

## ETHICS STATEMENT

This article does not contain any studies with human or animal materials performed by any of the authors.

## Data Availability

Data sharing is not applicable to this article.
